# Author Correction: The widespread and unjust drinking water and clean water crisis in the United States

**DOI:** 10.1038/s41467-023-38060-0

**Published:** 2023-06-13

**Authors:** J. Tom Mueller, Stephen Gasteyer

**Affiliations:** 1 Department of Sociology, Social Work, and Anthropology, Utah State University, Logan, UT USA; 2 Department of Sociology, Michigan State University, East Lansing, MI USA

**Keywords:** Sociology, Water resources

Correction to: *Nature Communications* 10.1038/s41467-021-23898-z, published online 22 June 2021

The original version of this Article contained incorrect data for thirteen US states in relation to the “Clean Water Act”. This data has been redacted from the study, and three additional sensitivity analysis tests performed to account for the omission. As a result of the additional sensitivity tests, reported results as pertains directly to the Clean Water Act have been updated in Table 1, Table 2, Table 3, Table 4, and Figure 4. Figure 3, which shows a map of the percent of county Clean Water Act (CWA) permittees listed as Clean Water Act Significant Noncompliers, has been updated to omit the thirteen states with incorrect data. Values in Supplementary Table 4 have been updated to reflect new results. Discussion and mention of the Clean Water Act data and results and also been updated throughout the main text file to account for these changes. An acknowledgement has been added to the individual (Colleen Keltz) who alerted authors to issues with the Clean Water Act Data. This has been corrected in the PDF and HTML versions of the Article.

## Supplementary information

Updated Supplementary Information

