# Addendum: The widespread and unjust drinking water and clean water crisis in the United States

**DOI:** 10.1038/s41467-023-38062-y

**Published:** 2023-06-13

**Authors:** J. Tom Mueller, Stephen Gasteyer

**Affiliations:** 1 Department of Geography and Environmental Sustainability, University of Oklahoma, Norman, OK USA; 2 Department of Sociology, Michigan State University, East Lansing, MI USA

## Background

In the initial version of our paper, we included Clean Water Act data for all 50 states, DC, and Puerto Rico. However, upon publication we were alerted to the fact that 13 states had data issues impacting the accuracy of the Clean Water Act data. These states include Iowa, Kansas, Michigan, Missouri, Nebraska, North Carolina, Ohio, Pennsylvania, Vermont, Washington, West Virginia, Wisconsin, and Wyoming. The exact issue varies from state to state, but in general it means that these states appear to have far more Clean Water Act permittees in Significant Noncompliance than there actually are (see https://echo.epa.gov/resources/echo-data/known-data-problems for an up to date description of all known data issues with Enforcement and Compliance History Online data). To ensure that we did not misrepresent the level of Clean Water Act Significant Noncompliance in these states, we have corrected our article by removing these states from the Clean Water Act portion of our analysis. Furthermore, we have now added a series of sensitivity tests where we estimate the scope of Clean Water Act Significant Noncompliance and model the injustice associated with elevated levels of this issue under two additional scenarios. The first includes all counties in the 50 states, DC, and Puerto Rico—the two non-state entities with available data—and is the same as our initially published version of this paper; and the second replaces the counties lost when dropping problem states by duplicating the top and bottom 20% of counties in the remaining pool of counties when problem states are removed. This duplication allowed us to generate plausible estimates of the overall scope of the issue by making the assumption that the removed counties (which were equal to 40% of the remaining counties) were split between very high or very low levels of Significant Noncompliance.

All results for the portions of our paper on incomplete plumbing and Safe Drinking Water Act Serious Violators are unchanged. Although we did not alter our analysis of Safe Drinking Water Act data, it should be noted that the EPA does report an unspecified number of inaccuracies and underreporting issues in their data for drinking water, as well as a small number of community water systems in Washington that may be inaccurate—which we did not note in our initial manuscript. These issues for Washington do not rise to the level of a “Primary Data Alert” and we thus elected to retain Washington for that portion of our analysis. To acknowledge these issues, we edited our Methods section and note that our analysis of Safe Drinking Water Act data reflects drinking water quality “as reported by the EPA” in August of 2020, which may contain some inaccuracies.

## Results

When we compare our corrected results with our original results/first sensitivity test scenario, our national estimates of the number of permittees in Significant Noncompliance are, unsurprisingly, impacted. When we remove the 13 issue states, our estimate of the number of permittees in Significant Noncompliance drops from 21,035 to 9457. Similarly, the percent of Clean Water Act permittees in Significant Noncompliance drops from 6.01 to 3.37% and the average percent of permittees in Significant Noncompliance drops from 9.00 to 6.23%. Although the number of counties with greater than 1% of Clean Water Act permittees in Significant Noncompliance drops from 2178 to 1455, the percent of counties with elevated levels of this issue only drops from 67.91 to 64.32%. When we map this issue, the regionality of the issue is not noticeably altered except for the fact that we now cannot assess Clean Water Act Significant Noncompliance in 13 states.

When we compare our corrected results with our second scenario where we duplicate cases, the differences are not as stark as they were when using the complete EPA data. For example, the total percent of permittees in violation only rises from 3.37 to 3.87% and the percent of counties with elevated levels of Clean Water Act Significant Noncompliance actually dropped from 64.32 to 59.81%. This result, wherein the increase between the primary estimates and the duplication estimates are not as stark as between the primary estimates and the estimates with all EPA data included, is in line with expectations since the majority of the data issues noted by the EPA are cases where there is overreporting of Significant Noncompliance.

Our models of elevated levels of Clean Water Act Significant Noncompliance—meaning greater than 1% of permittees in Significant Noncompliance—do not appreciably change when we remove the 13 issue states, nor when we conduct the duplication analysis (Fig. 1). The only notable changes are that percent Latino/a is statistically significant at *p* < 0.05 in the pure descriptive model of the primary results and not in the results with all counties included; and percent without a high school diploma is significant and percent Latino/a is not in the full model of the duplication results. These changes highlight the dubious nature of the threshold of *p* < 0.05, as the *p* values of these coefficients were very near the 0.05 threshold. For example, in the original paper the *p* value for Latino/a was 0.073 in the descriptive model and in the corrected paper it is now 0.018. This threshold effect is visually demonstrated in Fig. 1, where we compare the results between all three specifications. As can be seen, the modeling results, and subsequent takeaways are not impacted by the removal of the 13 issue states or the replacement of missing counties via duplication.

**Fig. 1:**
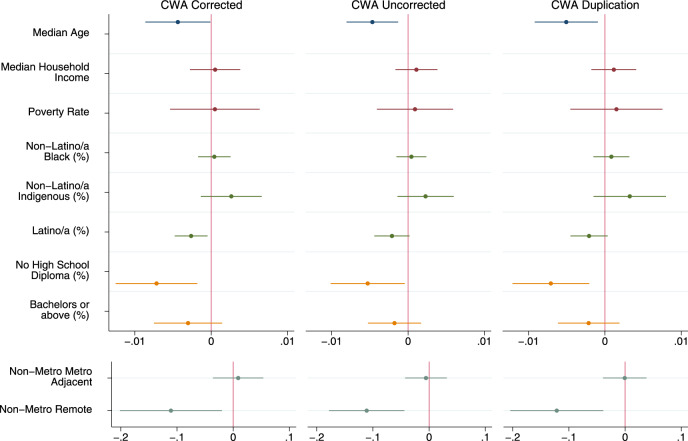
Coefficient plot of Clean Water Act sensitivity test results. Descriptive regression model results. Different colors for plotted coefficients represent separate blocks of variables. Models are linear probability models with state fixed effects and Huber/White/Sandwich cluster-robust standard errors at the state level. All tests two-tailed. Dots indicate point estimates and lines represent 95% confidence intervals. Models predicted whether or not there were greater than 1% of Clean Water Act permittees being considered in Significant Noncompliance in the county. First model excludes counties in states with CWA data issues (*N* = 2261), second model includes all counties reported by the EPA (*N* = 3206), and third model duplicates counties in the top and bottom 10% of CWA Significant Noncompliance within states without data issues (*N* = 3151). Full model results, confidence intervals, and exact *p* values available in the SI of the corrected article.

## Summary

In sum, this correction does not change the core takeaway of our paper, and in many ways amplifies a point made in the original article regarding EPA data quality. When we drop the 13 states with data issues, there are still millions of Americans—153,686,279—living in counties with elevated levels of Clean Water Act Significant Noncompliance, and our models still show that there is far more evidence of injustice related to incomplete plumbing than water quality. Furthermore, the fact that EPA Clean Water Act data are unreliable for over a fifth of the US states, and that Safe Drinking Water Act data are possibly inaccurate for an unspecified number of community water systems, bolsters our initial critique of the quality and usability of federal data on the unaddressed household water crisis in the United States.

