# Supplementary material for: The widespread and unjust drinking water and clean water crisis in the United States
Source: Nat Commun. 2021 Jun 22;12:3544. doi: 10.1038/s41467-021-23898-z (PMC8219686; doi:10.1038/s41467-021-23898-z)
Supplement: Supplementary file 1 — Supplementary Information [file 41467_2021_23898_MOESM1_ESM.docx]

**Supplementary Information File**

**The Widespread Unjust Drinking Water and Clean Water Crisis in the United States**

**Supplementary Table 1.** Summary Statistics for Model Variables

|  | Mean | SD | Min | Max |
| --- | --- | --- | --- | --- |
| Median Age | 41.28 | 5.36 | 21.70 | 67.00 |
| Median Household Income | 50.79 | 14.46 | 12.81 | 136.27 |
| Poverty Rate | 16.39 | 8.23 | 2.30 | 64.16 |
| Percent Black | 8.71 | 14.35 | 0.00 | 87.41 |
| Percent Indigenous | 1.87 | 7.50 | 0.00 | 90.99 |
| Percent Hispanic | 11.43 | 19.37 | 0.00 | 100.00 |
| Percent without High School Diploma | 13.75 | 6.68 | 1.18 | 66.34 |
| Percent with at least a Bachelor's Degree | 21.57 | 9.36 | 0.00 | 78.53 |
| Median Age | 41.28 | 5.36 | 21.70 | 67.00 |
| *N* | 3220 |  |  |  |

Note: For the three-category indicator of rurality there were 1235 counties classified as metropolitan, 1034 counties classified as non-metropolitan metropolitan-adjacent, and 951 counties classified as non-metropolitan remote.

**Supplementary Table 2.** Social Factors Associated with Elevated Levels of Incomplete Plumbing

|  | Age | Income | Race/Ethnicity | Education | Rurality | Full Model |
| --- | --- | --- | --- | --- | --- | --- |
| Median Age | **0.0083 (0.000)** |  |  |  |  | **0.0105 (0.000)** |
|  | [0.0047,0.0119] |  |  |  |  | [0.0068,0.0141] |
| Median Household Income |  | **-0.0036 (0.000)** |  |  |  | 0.0008 (0.539) |
|  |  | [-0.0055,-0.0017] |  |  |  | [-0.0017,0.0032] |
| Poverty Rate |  | **0.0061 (0.005)** |  |  |  | **0.0098 (0.001)** |
|  |  | [0.0019,0.0103] |  |  |  | [0.0042,0.0154] |
| Percent Non-Latino/a Black |  |  | -0.0004 (0.685) |  |  | -0.0015 (0.057) |
|  |  |  | [-0.0024,0.0016] |  |  | [-0.0030,0.0000] |
| Percent Non-Latino/a Indigenous |  |  | **0.0055 (0.000)** |  |  | **0.0034 (0.049)** |
|  |  |  | [0.0027,0.0084] |  |  | [0.0000,0.0067] |
| Percent Latino/a |  |  | 0.0001 (0.955) |  |  | -0.0001 (0.950) |
|  |  |  | [-0.0048,0.0050] |  |  | [-0.0046,0.0043] |
| Percent without High School Diploma |  |  |  | **0.0069 (0.012)** |  | **0.0061 (0.026)** |
|  |  |  |  | [0.0016,0.0123] |  | [0.0008,0.0113] |
| Percent with at least a Bachelor's Degree |  |  |  | **-0.0044 (0.000)** |  | -0.0003 (0.773) |
|  |  |  |  | [-0.0066,-0.0023] |  | [-0.0026,0.0020] |
| Metro Status (Metro = Reference) | | |  |  |  |  |
| Non-Metro Metro-Adjacent |  |  |  |  | **0.1067 (0.000)** | 0.0295 (0.075) |
|  |  |  |  |  | [0.0743,0.1390] | [-0.0029,0.0620] |
| Non-Metro Remote |  |  |  |  | **0.1402 (0.000)** | **0.0491 (0.034)** |
|  |  |  |  |  | [0.1034,0.1770] | [0.0042,0.0941] |
| Constant | -0.1844 (0.016) | 0.2420 (0.003) | 0.1498 (0.000) | 0.1582 (0.006) | 0.0824 (0.000) | -0.5652 (0.005) |
|  | [-0.3337,-0.0352] | [0.0856,0.3984] | [0.0927,0.2068] | [0.0476,0.2687] | [0.0670,0.0978] | [-0.7960,-0.3344] |
| *N* | 3220 | 3219 | 3220 | 3220 | 3220 | 3219 |

Unstandardized coefficients on left and exact *p*-values in parentheses. 95% confidence intervals in brackets. Coefficients with *p*<.05 bolded. Note: All models are linear probability models where the dependent variable is a dichotomous classification of whether or not greater than 1.0% of the county had incomplete access to plumbing. State level fixed effects included in model estimation and robust standard errors used. Condition Index = 5.48 and all VIF<10. Individual coefficients evaluated via two-tailed *t*-test and no adjustments to *p*-values were made for multiple comparisons.

**Supplementary Table 3.** Social Factors Associated with Elevated Levels of Safe Drinking Water Act Serious Violators

|  | Age | Income | Race/Ethnicity | Education | Rurality | Full Model |
| --- | --- | --- | --- | --- | --- | --- |
| Median Age | -0.0023 (0.132) |  |  |  |  | 0.0010 (0.663) |
|  | [-0.0053,0.0007] |  |  |  |  | [-0.0034,0.0054] |
| Median Household Income |  | **0.0026 (0.001)** |  |  |  | 0.0013 (0.242) |
|  |  | [0.0010,0.0041] |  |  |  | [-0.0009,0.0034] |
| Poverty Rate |  | **0.0065 (0.001)** |  |  |  | **0.0067 (0.002)** |
|  |  | [0.0028,0.0102] |  |  |  | [0.0026,0.0109] |
| Percent Non-Latino/a Black |  |  | 0.0011 (0.139) |  |  | 0.0002 (0.737) |
|  |  |  | [-0.0004,0.0026] |  |  | [-0.0011,0.0015] |
| Percent Non-Latino/a Indigenous |  |  | 0.0017 (0.166) |  |  | 0.0004 (0.769) |
|  |  |  | [-0.0008,0.0042] |  |  | [-0.0025,0.0034] |
| Percent Latino/a |  |  | -0.0002 (0.873) |  |  | -0.0001 (0.970) |
|  |  |  | [-0.0033,0.0029] |  |  | [-0.0045,0.0044] |
| Percent without High School Diploma |  |  |  | -0.0002 (0.929) |  | -0.0021 (0.400) |
|  |  |  |  | [-0.0047,0.0043] |  | [-0.0070,0.0029] |
| Percent with at least a Bachelor's Degree |  |  |  | 0.0014 (0.163) |  | 0.0006 (0.662) |
|  |  |  |  | [-0.0006,0.0033] |  | [-0.0020,0.0031] |
| Metro Status (Metro = Reference) | | |  |  |  |  |
| Non-Metro Metro-Adjacent |  |  |  |  | -0.0133 (0.421) | -0.0135 (0.521) |
|  |  |  |  |  | [-0.0462,0.0196] | [-0.0555,0.0284] |
| Non-Metro Remote |  |  |  |  | **-0.0738 (0.015)** | **-0.0727 (0.022)** |
|  |  |  |  |  | [-0.1327,-0.0149] | [-0.1345,-0.0109] |
| Constant | 0.2837 (0.000) | -0.0458 (0.482) | 0.1792 (0.000) | 0.1628 (0.001) | 0.2160 (0.000) | 0.0181 (0.900) |
|  | [0.1602,0.4072] | [-0.1754,0.0839] | [0.1499,0.2086] | [0.0690,0.2567] | [0.1897,0.2423] | [-0.2692,0.3055] |
| *N* | 3144 | 3143 | 3144 | 3144 | 3144 | 3143 |

Unstandardized coefficients on left and exact *p*-values in parentheses. 95% confidence intervals in brackets. Coefficients with *p*<.05 bolded.

Note: All models are linear probability models where the dependent variable is a dichotomous classification of whether or not greater than 1.0% of the county CWS's were listed as a SDWA Significant Violator. State level fixed effects included in model estimation and robust standard errors used. Condition Index = 5.48 and all VIF<10. Individual coefficients evaluated via two-tailed *t*-test and no adjustments to *p*-values were made for multiple comparisons.

**Supplementary Table 4.** Social Factors Associated with Elevated Levels of Clean Water Act Significant Noncompliance – Primary Models

|  | Age | Income | Race/Ethnicity | Education | Rurality | Full Model |
| --- | --- | --- | --- | --- | --- | --- |
| Median Age | **-0.0044 (0.045)** |  |  |  |  | **-0.0072 (0.006)** |
|  | [-0.0086,-0.0001] |  |  |  |  | [-0.0122,-0.0022] |
| Median Household Income |  | 0.0005 (0.754) |  |  |  | -0.0004 (0.850) |
|  |  | [-0.0028,0.0038] |  |  |  | [-0.0052,0.0043] |
| Poverty Rate |  | 0.0005 (0.866) |  |  |  | -0.0015 (0.689) |
|  |  | [-0.0054,0.0064] |  |  |  | [-0.0088,0.0059] |
| Percent Non-Latino/a Black |  |  | 0.0004 (0.698) |  |  | 0.0001 (0.900) |
|  |  |  | [-0.0017,0.0025] |  |  | [-0.0015,0.0017] |
| Percent Non-Latino/a Indigenous |  |  | 0.0026 (0.189) |  |  | 0.0018 (0.443) |
|  |  |  | [-0.0014,0.0066] |  |  | [-0.0028,0.0063] |
| Percent Latino/a |  |  | **-0.0026 (0.018)** |  |  | **-0.0029 (0.045)** |
|  |  |  | [-0.0048,-0.0005] |  |  | [-0.0057,-0.0001] |
| Percent without High School Diploma |  |  |  | **-0.0072 (0.010)** |  | -0.0046 (0.118) |
|  |  |  |  | [-0.0125,-0.0018] |  | [-0.0104,0.0012] |
| Percent with at least a Bachelor's Degree |  |  |  | -0.0030 (0.178) |  | -0.0035 (0.171) |
|  |  |  |  | [-0.0075,0.0014] |  | [-0.0085,0.0016] |
| Metro Status (Metro = Reference) | | |  |  |  |  |
| Non-Metro Metro-Adjacent |  |  |  |  | 0.0089 (0.690) | 0.0118 (0.639) |
|  |  |  |  |  | [-0.0358,0.0536] | [-0.0385,0.0621] |
| Non-Metro Remote |  |  |  |  | **-0.1108 (0.018)** | **-0.0985 (0.043)** |
|  |  |  |  |  | [-0.2015,-0.0201] | [-0.1936,-0.0035] |
| Constant | 0.8213 (0.000) | 0.6086 (0.000) | 0.6698 (0.000) | 0.8149 (0.000) | 0.6710 (0.000) | 1.1857 (0.000) |
|  | [0.6475,0.9951] | [0.3513,0.8659] | [0.6398,0.6999] | [0.6524,0.9774] | [0.6343,0.7077] | [0.7191,1.6524] |
| *N* | 2262 | 2261 | 2262 | 2262 | 2262 | 2261 |

Unstandardized coefficients on left and exact *p*-values in parentheses. 95% confidence intervals in brackets. Coefficients with *p*<.05 bolded. Note: All models are linear probability models where the dependent variable is a dichotomous classification of whether or not greater than 1.0% of the county CWA permittees were listed as CWA Significant Noncompliers. State level fixed effects included in model estimation and robust standard errors used. Condition Index = 5.63 and all VIF<10. Individual coefficients evaluated via two-tailed *t*-test and no adjustments to *p*-values were made for multiple comparisons. The 13 states with CWA data issues are not included in models.

**Supplementary Table 5.** Social Factors Associated with Elevated Levels of Clean Water Act Significant Noncompliance – All Counties

|  | Age | Income | Race/Ethnicity | Education | Rurality | Full Model |
| --- | --- | --- | --- | --- | --- | --- |
| Median Age | **-0.0047 (0.008)** |  |  |  |  | **-0.0065 (0.002)** |
|  | [-0.0081,-0.0013] |  |  |  |  | [-0.0106,-0.0025] |
| Median Household Income |  | 0.0011 (0.426) |  |  |  | -0.0002 (0.921) |
|  |  | [-0.0017,0.0039] |  |  |  | [-0.0041,0.0037] |
| Poverty Rate |  | 0.0009 (0.714) |  |  |  | -0.0016 (0.601) |
|  |  | [-0.0041,0.0059] |  |  |  | [-0.0079,0.0046] |
| Percent Non-Latino/a Black |  |  | 0.0004 (0.655) |  |  | 0.0001 (0.905) |
|  |  |  | [-0.0015,0.0024] |  |  | [-0.0015,0.0017] |
| Percent Non-Latino/a Indigenous |  |  | 0.0023 (0.216) |  |  | 0.0016 (0.435) |
|  |  |  | [-0.0014,0.0060] |  |  | [-0.0025,0.0056] |
| Percent Latino/a |  |  | -0.0021 (0.073) |  |  | **-0.0027 (0.041)** |
|  |  |  | [-0.0044,0.0002] |  |  | [-0.0053,-0.0001] |
| Percent without High School Diploma |  |  |  | **-0.0053 (0.033)** |  | -0.0029 (0.231) |
|  |  |  |  | [-0.0101,-0.0004] |  | [-0.0077,0.0019] |
| Percent with at least a Bachelor's Degree |  |  |  | -0.0018 (0.310) |  | -0.0030 (0.122) |
|  |  |  |  | [-0.0053,0.0017] |  | [-0.0068,0.0008] |
| Metro Status (Metro = Reference) | | |  |  |  |  |
| Non-Metro Metro-Adjacent |  |  |  |  | -0.0056 (0.762) | -0.0040 (0.841) |
|  |  |  |  |  | [-0.0427,0.0315] | [-0.0438,0.0358] |
| Non-Metro Remote |  |  |  |  | **-0.1111 (0.002)** | **-0.0985 (0.006)** |
|  |  |  |  |  | [-0.1781,-0.0440] | [-0.1682,-0.0288] |
| Constant | 0.8721 (0.000) | 0.6082 (0.000) | 0.6951 (0.000) | 0.7901 (0.000) | 0.7136 (0.000) | 1.1485 (0.000) |
|  | [0.7326,1.0115] | [0.3962,0.8202] | [0.6701,0.7202] | [0.6587,0.9216] | [0.6854,0.7418] | [0.7597,1.5373] |
| *N* | 3207 | 3206 | 3207 | 3207 | 3207 | 3206 |

Unstandardized coefficients on left and exact *p*-values in parentheses. 95% confidence intervals in brackets. Coefficients with *p*<.05 bolded. Note: All models are linear probability models where the dependent variable is a dichotomous classification of whether or not greater than 1.0% of the county CWA permittees were listed as CWA Significant Noncompliers. State level fixed effects included in model estimation and robust standard errors used. Condition Index = 5.48 and all VIF<10. Individual coefficients evaluated via two-tailed *t*-test and no adjustments to *p*-values were made for multiple comparisons. All counties in the 50 US states, DC, and Puerto Rico included—regardless of EPA data flag.

**Supplementary Table 6.** Social Factors Associated with Elevated Levels of Clean Water Act Significant Noncompliance – Duplication Test

|  | Age | Income | Race/Ethnicity | Education | Rurality | Full Model |
| --- | --- | --- | --- | --- | --- | --- |
| Median Age | **-0.0051 (0.018)** |  |  |  |  | **-0.0063 (0.019)** |
|  | [-0.0092,-0.0009] |  |  |  |  | [-0.0115,-0.0011] |
| Median Household Income |  | 0.0012 (0.428) |  |  |  | -0.0008 (0.745) |
|  |  | [-0.0018,0.0041] |  |  |  | [-0.0057,0.0041] |
| Poverty Rate |  | 0.0015 (0.617) |  |  |  | -0.0011 (0.775) |
|  |  | [-0.0045,0.0075] |  |  |  | [-0.0087,0.0065] |
| Percent Non-Latino/a Black |  |  | 0.0008 (0.473) |  |  | 0.0007 (0.481) |
|  |  |  | [-0.0015,0.0032] |  |  | [-0.0012,0.0026] |
| Percent Non-Latino/a Indigenous |  |  | 0.0033 (0.173) |  |  | 0.0027 (0.327) |
|  |  |  | [-0.0015,0.0080] |  |  | [-0.0028,0.0082] |
| Percent Latino/a |  |  | -0.0021 (0.093) |  |  | -0.0016 (0.313) |
|  |  |  | [-0.0045,0.0004] |  |  | [-0.0047,0.0016] |
| Percent without High School Diploma |  |  |  | **-0.0071 (0.007)** |  | **-0.0066 (0.023)** |
|  |  |  |  | [-0.0121,-0.0021] |  | [-0.0122,-0.0010] |
| Percent with at least a Bachelor's Degree |  |  |  | -0.0021 (0.293) |  | -0.0026 (0.275) |
|  |  |  |  | [-0.0062,0.0019] |  | [-0.0073,0.0021] |
| Metro Status (Metro = Reference) | | |  |  |  |  |
| Non-Metro Metro-Adjacent |  |  |  |  | -0.0006 (0.976) | 0.0099 (0.718) |
|  |  |  |  |  | [-0.0391,0.0380] | [-0.0451,0.0648] |
| Non-Metro Remote |  |  |  |  | **-0.1216 (0.005)** | -**0.1007 (0.044)** |
|  |  |  |  |  | [-0.2042,-0.0389] | [-0.1983,-0.0030] |
| Constant | 0.8055 (0.000) | 0.5132 (0.000) | 0.6117 (0.000) | 0.7502 (0.000) | 0.6353 (0.000) | 1.1060 (0.000) |
|  | [0.6361,0.9749] | [0.2729,0.7535] | [0.5772,0.6462] | [0.6024,0.8981] | [0.6013,0.6693] | [0.6248,1.5873] |
| *N* | 3153 | 3151 | 3153 | 3153 | 3153 | 3151 |

Unstandardized coefficients on left and exact *p*-values in parentheses. 95% confidence intervals in brackets. Coefficients with *p*<.05 bolded. Note: All models are linear probability models where the dependent variable is a dichotomous classification of whether or not greater than 1.0% of the county CWA permittees were listed as CWA Significant Noncompliers. State level fixed effects included in model estimation and robust standard errors used. Condition Index = 5.64 and all VIF<10. Individual coefficients evaluated via two-tailed *t*-test and no adjustments to *p*-values were made for multiple comparisons. Counties with the top and bottom 20% of Significant Noncompliance duplicated for analysis.

**Supplemental Figures**

**

**

**Supplementary Fig. 1.** Map of the percent of county Clean Water Act (CWA) permittees listed as Clean Water Act Significant Noncompliers. All facilities that discharge directly into water of the United States are issued a Clean Water Act permit, those who represent a more severe level of environmental threat due to violations and noncompliance are considered in Significant Noncompliance. Map includes all data as reported by the EPA—including the thirteen states with known data issues.
