# Supplementary material for: The widespread and unjust drinking water and clean water crisis in the United States
Source: Nat Commun. 2021 Jun 22;12:3544. doi: 10.1038/s41467-021-23898-z (PMC8219686; doi:10.1038/s41467-021-23898-z)
Supplement: Supplementary file 3 — Reporting Summary [file 41467_2021_23898_MOESM3_ESM.pdf]

nature research

Corresponding author(s): J. Tom Mueller

Last updated by author(s): 4/22/2021

Reporting Summary

Nature Research wishes to improve the reproducibility of the work that we publish. This form provides structure for consistency and transparency in reporting. For further information on Nature Research policies, see our Editorial Policies and the Editorial Policy Checklist.

Statistics

For all statistical analyses, confirm that the following items are present in the figure legend, table legend, main text, or Methods section.

n/a	Confirmed	
☐	☒	The exact sample size (*n*) for each experimental group/condition, given as a discrete number and unit of measurement
☐	☒	A statement on whether measurements were taken from distinct samples or whether the same sample was measured repeatedly
☐	☒	The statistical test(s) used AND whether they are one- or two-sided<br>*Only common tests should be described solely by name; describe more complex techniques in the Methods section.*
☐	☒	A description of all covariates tested
☐	☒	A description of any assumptions or corrections, such as tests of normality and adjustment for multiple comparisons
☐	☒	A full description of the statistical parameters including central tendency (e.g. means) or other basic estimates (e.g. regression coefficient) AND variation (e.g. standard deviation) or associated estimates of uncertainty (e.g. confidence intervals)
☐	☒	For null hypothesis testing, the test statistic (e.g. *F*, *t*, *r*) with confidence intervals, effect sizes, degrees of freedom and *P* value noted<br>*Give P values as exact values whenever suitable.*
☒	☐	For Bayesian analysis, information on the choice of priors and Markov chain Monte Carlo settings
☒	☐	For hierarchical and complex designs, identification of the appropriate level for tests and full reporting of outcomes
☒	☐	Estimates of effect sizes (e.g. Cohen's *d*, Pearson's *r*), indicating how they were calculated

Our web collection on statistics for biologists contains articles on many of the points above.

Software and code

Policy information about availability of computer code

Data collection	Data was collated in Stata 16.0. All code was purpose-written for this study. The data was manually downloaded from the public data repositories listed in the manuscript. All code is available and posted on the OSF project for this paper located at DOI 10.17605/OSF.IO/ZPQR9.
Data analysis	Data was analyzed in Stata 16.0 and mapped in QGIS 3.10. All code for this analysis was purpose-written. All code is available and posted on the OSF project for this paper located at DOI 10.17605/OSF.IO/ZPQR9.

For manuscripts utilizing custom algorithms or software that are central to the research but not yet described in published literature, software must be made available to editors and reviewers. We strongly encourage code deposition in a community repository (e.g. GitHub). See the Nature Research guidelines for submitting code & software for further information.

Data

Policy information about availability of data

All manuscripts must include a data availability statement. This statement should provide the following information, where applicable:

- Accession codes, unique identifiers, or web links for publicly available datasets
- A list of figures that have associated raw data
- A description of any restrictions on data availability

Data for this study are available on the OSF project for this paper located at DOI 10.17605/OSF.IO/ZPQR9. All figures in the manuscript have associated raw data which is generated via this code. All data is available for replication.

Field-specific reporting

Please select the one below that is the best fit for your research. If you are not sure, read the appropriate sections before making your selection.

☐ Life sciences ☒ Behavioural & social sciences ☐ Ecological, evolutionary & environmental sciences

For a reference copy of the document with all sections, see nature.com/documents/nr-reporting-summary-flat.pdf

Behavioural & social sciences study design

All studies must disclose on these points even when the disclosure is negative.

Study description	This study is a quantitative analysis of the prevalence of water hardship in the United States. The dependent variables include prevalence of incomplete plumbing, prevalence of Safe Drinking Water Act violations, and prevalence of Clean Water Act violations. The analysis includes descriptive statistics and mapping, as well as multiple regressions of sociodemographic correlates of these issues.
Research sample	All counties in the United States of America are included. The data was generated via the sampling strategy of the American Community Survey, which is generalizable to each county in the United States when using five-year estimations. The sampling rationale follows best practices from the U.S. Census Bureau.
Sampling strategy	The sampling used to generate our estimates of incomplete plumbing and sociodemographics is conducted by the US Census Bureau via the American Community Survey (ACS). The water quality data is complete data from the EPA. The determination to use two datasets was made because it was necessary to allow us to ask our research questions regarding both incomplete plumbing (ACS) and water quality (EPA).
Data collection	Data was collected by downloading the American Community Survey estimates from IPUMS-NHGIS and the ECHO database hosted by the EPA. The data from the census was anonymized and aggregated before the researchers extracted the data.
Timing	The most recent appropriate ACS data was collected from IPUMS-NHGIS (2014-2018), the EPA data was extracted on August 18, 2020 and represents that point in time.
Data exclusions	As detailed in the methods, a small number of water utilities were excluded due to a lack of geographic data (10 for Clean Water Act data and 1,334 for Safe Drinking Water Act data). Further, for each type of water quality a small portion of counties had no utilities, meaning those counties were excluded (13 for Clean Water Act and 76 for Safe Drinking Water Act data). All other data was included.
Non-participation	As this study used aggregate data provided via the U.S. Census Bureau and the EPA, non-participation was not applicable to this analysis.
Randomization	As this study was observational and non-experimental, randomization was not applicable to this study.

Reporting for specific materials, systems and methods

We require information from authors about some types of materials, experimental systems and methods used in many studies. Here, indicate whether each material, system or method listed is relevant to your study. If you are not sure if a list item applies to your research, read the appropriate section before selecting a response.

Materials & experimental systems

n/a	Involved in the study	
☒	☐	Antibodies
☒	☐	Eukaryotic cell lines
☒	☐	Palaeontology and archaeology
☒	☐	Animals and other organisms
☒	☐	Human research participants
☒	☐	Clinical data
☒	☐	Dual use research of concern

Methods

n/a	Involved in the study	
☒	☐	ChIP-seq
☒	☐	Flow cytometry
☒	☐	MRI-based neuroimaging
